# The Incidence of Bone Cement Implantation Syndrome Grade 3 in Hip and Knee Arthroplasty: A Retrospective Multicenter Case Series

**DOI:** 10.2106/JBJS.OA.26.00252

**Published:** 2026-09-22

**Authors:** Karen E. Brokke, Sjoerd Servaas, Inger N. Sierevelt, Karel W.J. Klop, Sef J.A. van Bilsen, Laurens Kaas, Loes W.A.H. van Beers, Erik H.P.A. van Dongen, Rutger van Geenen, W. Anton Visser, Iris Koenraadt-van Oost, Monique A.H. Steegers, Peter A. Nolte

**Affiliations:** 1 Department of Orthopaedic Surgery, Spaarne Gasthuis Academy, Hoofddorp, The Netherlands; 2 Department of Anaesthesia, Amsterdam Movement Sciences, Amsterdam University Medical Centers, Amsterdam, The Netherlands; 3 Department of Anaesthesia, Spaarne Gasthuis Academy, Hoofddorp, The Netherlands; 4 Department of Orthopaedic Surgery and Sports Medicine, Amsterdam Movement Sciences, Amsterdam University Medical Centers, Amsterdam, The Netherlands; 5 Department of Orthopaedic Surgery, Xpert Clinics, Amsterdam, The Netherlands; 6 Department of Surgery, Spaarne Gasthuis Academy, Hoofddorp, The Netherlands; 7 Department of Anaesthesia, Elisabeth-Tweesteden Hospital, Tilburg, The Netherlands; 8 Department of Orthopaedic Surgery, St. Antonius Hospital, Utrecht, The Netherlands; 9 Department of Anaesthesia, St. Antonius Hospital, Utrecht, The Netherlands; 10 Department of Orthopaedic Surgery, Amphia Hospital, Breda, The Netherlands; 11 Department of Anaesthesia, Amphia Hospital, Breda, The Netherlands; 12 Foundation for Orthopaedic Research, Care & Education Amphia Hospital, Breda, The Netherlands; 13 Department of Oral Cell Biology, Academic Centre for Dentistry, University of Amsterdam and Vrije Universiteit Amsterdam, Amsterdam, The Netherlands

## Abstract

**Background:**

Cardiac arrest during hip or knee arthroplasty (KA) represents the most severe manifestation of bone cement implantation syndrome (BCIS). However, true incidence rates remain uncertain, and data on clinical characteristics and outcomes are limited. The aim of this study was to determine the incidence of BCIS grade 3 in hip and KA and to describe associated clinical characteristics and mortality.

**Methods:**

This retrospective multicenter case series included all hip and KA procedures performed at 4 Dutch hospitals. Potential cases were identified through database queries. Medical records were manually reviewed to confirm BCIS grade 3, defined as cardiovascular collapse requiring cardiopulmonary resuscitation occurring from joint manipulation through skin closure.

**Results:**

No cases of BCIS grade 3 occurred during 25,914 knee arthroplasties. Among 39,237 hip procedures, 31 patients experienced BCIS grade 3 (0.08%), of which 27 cases occurred during hemiarthroplasty for femoral neck fracture (FNF). The mean age of patients with BCIS grade 3 was 86.2 ± 7.7 years; 71.0% were female, 93.5% underwent acute surgery for FNF, 87.1% involved cemented procedures, and perioperative mortality was 67.7%.

**Conclusion:**

BCIS grade 3 is rare but highly lethal, occurring predominantly during hemiarthroplasty for FNF in elderly patients. Individualized risk assessment and informed consent are essential when managing FNF in high-risk patients.

**Level of Evidence:**

Prognostic Level IV. See Instructions for Authors for a complete description of levels of evidence.

## Introduction

Intraoperative cardiac arrest (IOCA) is a rare but potentially fatal complication during hip and knee arthroplasty (KA) and is considered the most severe manifestation of bone cement implantation syndrome (BCIS)^1^. This syndrome is characterized by hypoxia, hypotension, loss of consciousness, and/or cardiac arrest occurring in temporal relation with cementation or prosthesis insertion^1,2^. Donaldson et al. classified BCIS into 3 grades, with grade 3 defined as cardiovascular collapse requiring cardiopulmonary resuscitation^1^.

The etiology of BCIS is considered multifactorial and likely involves embolic and inflammatory mechanisms^1^. Although the term “bone cement implantation syndrome” implies a causal relationship with cement use, recent evidence suggests that BCIS may also occur during procedures without cementation^1,2^. Therefore, it has been suggested that BCIS may be a misnomer^3^. Nevertheless, the term BCIS remains widely used in the literature.

The majority of the BCIS events present as grade 1 or 2^2^. Grade 3 remains rare and is primarily described in small cohort studies of hemiarthroplasty and total hip arthroplasty (THA), with reported incidences ranging from 0% to 3.4%^4-16^. Data regarding elective procedures, uncemented techniques, or KA are limited. Accurate incidence data are essential for informed consent, risk stratification, and the development of preventive strategies. Therefore, the aim of this study was to determine the incidence of BCIS grade 3 during cemented and uncemented hip and KA and to describe associated clinical characteristics and mortality.

## Method

### Study Design and Setting

This retrospective multicenter case series was conducted in 4 large Dutch nonacademic teaching hospitals (Spaarne Gasthuis Hospital, Hoofddorp; Elisabeth-TweeSteden Hospital, Tilburg; Amphia Hospital, Breda and St. Antonius Hospital, Utrecht). The study protocol was approved by the local review board of the Spaarne Gasthuis Hospital (2023.0083), and the need for written informed consent was waived due to the retrospective nature of the study.

Patients were included from implementation of the electronic medical record system (2008, 2016 or 2017) until 2023, 2024, or 2025, depending on the participating center.

### Procedural Details

All surgeries were performed as part of standard clinical care, with the type of anesthesia determined by the attending anesthesiologist. Bone cement was Optipac 60 Refobacin (Zimmer Biomet), Cemex System Genta Fast (Tecres S.p.A.), or PALACOS R+G pro (Heraeus Medical GmbH). In all cases, cement was mixed under vacuum and applied using a cement gun; finger packing was not used.

### Study Population

At each hospital, a structured database query was performed using procedural codes for cemented and uncemented KA (total and unicondylar), hemiarthroplasty, THA, and revision hip or KA, combined with codes for intraoperative death, in-hospital mortality, or intensive care unit (ICU) admission. Two independent reviewers (K.B. and S.S.) assessed all potential cases to determine whether cardiac arrest was consistent with BCIS. Cases were considered potentially BCIS related when cardiac arrest occurred in close temporal relation with joint manipulation, cementation, or prosthesis implantation and was preceded by acute hemodynamic deterioration without an alternative clear etiology. In cases of uncertainty, adjudication was performed through discussion with senior investigators (P.N., M.S.). Consensus was reached based on clinical presentation, timing, and plausibility of BCIS as the underlying cause.

BCIS grade 3 was defined as cardiovascular collapse requiring cardiopulmonary resuscitation (chest compressions) occurring at any point from initial joint manipulation through skin closure. This definition was extended to include cases requiring pharmacological resuscitation with vasopressor or inotropic agents administered in the setting of circulatory arrest, where these interventions were necessary to restore spontaneous circulation^1,2^. Exclusion criteria were as follows: no IOCA, IOCA occurring before joint manipulation or after skin closure, and IOCA with clear alternative etiology (e.g., documented myocardial infarction or pulmonary embolism).

### Data Collection

Baseline characteristics included age, sex, body mass index, smoking history, American Society of Anesthesiologists (ASA) classification, preoperative hemoglobin and creatinine levels, medical history, medication use, type of surgery, cement use, type of anesthesia, date of surgery, and date of death. Race and ethnicity are not included in the study.

A subanalysis was performed in patients undergoing hemiarthroplasty. Aggregated data on age, sex, and ASA classification were obtained from either EPIC (EPIC Systems Corporation) or the Dutch Arthroplasty Register (“Landelijke Registratie Orthopedische Interventies”, LROI). The LROI is a population-based registry that has been recording data on joint arthroplasties in The Netherlands since 2007. As reported completeness ranges from 50.0% to 99.0%^17^, we conducted an additional hospital-level data verification. In our study cohort, the proportion of missing data was between 5.3% and 15.5%.

### Statistical Analysis

All collected data were analyzed by use of IBM SPSS Statistics (version 26.0, IBM Corp.). Analysis was mainly descriptive. Continuous variables are described as means and SD, or medians with interquartile ranges in case of non-normality. Normality was assessed visually. Categorical variables are expressed as numbers with accompanying percentages. Survival rates were estimated using Kaplan-Meier survival analysis. A log transformation was performed to calculate the 95% confidence interval (CI).

A sensitivity analysis was performed because of concerns regarding underreporting at 1 hospital (10,566 procedures, only 2 identified grade 3 BCIS cases), despite an additional, more comprehensive review of the electronic medical records.

Reference values were calculated by performing a sample size–weighted pooling of the aggregated data from the participating hospitals. Comparisons of patients’ demographics were performed between patients with BCIS grade 3 and the reference group, and between patients who survived BCIS grade 3 vs. those who died perioperatively, using Student's *t*-tests (continuous variables) and chi-square or Fisher's exact tests (when expected cell counts were <5) for categorical variables. Owing to the explorative nature of the study, correction for multiple testing was not performed. A predefined threshold of 10% missing data was used to determine eligibility for statistical analysis; variables exceeding this threshold were excluded. This was based on the assumption of nonrandom missing data. Statistical significance was set at p < 0.05.

## Results

**Fig. 1:**
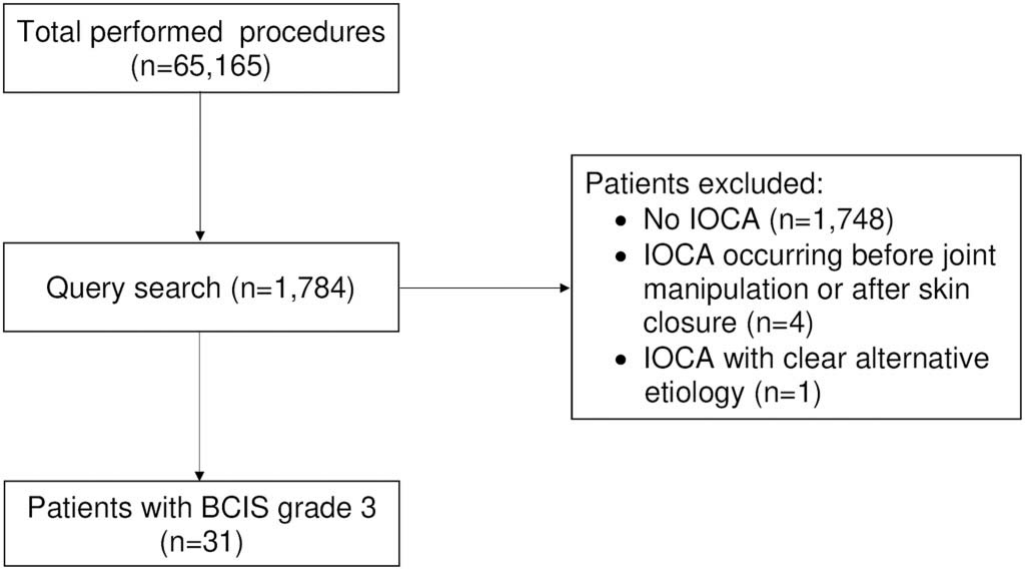
Flowchart. BCIS = bone cement implantation syndrome, and IOCA = intraoperative cardiac arrest.

A total of 65,151 procedures were identified, comprising 39,237 hip and 25,914 knee arthroplasties. Among the 39,237 hip procedures, 31 patients (0.08%) experienced BCIS grade 3 (Fig. 1). No cases were identified during 25,914 KA.

**Fig. 2:**
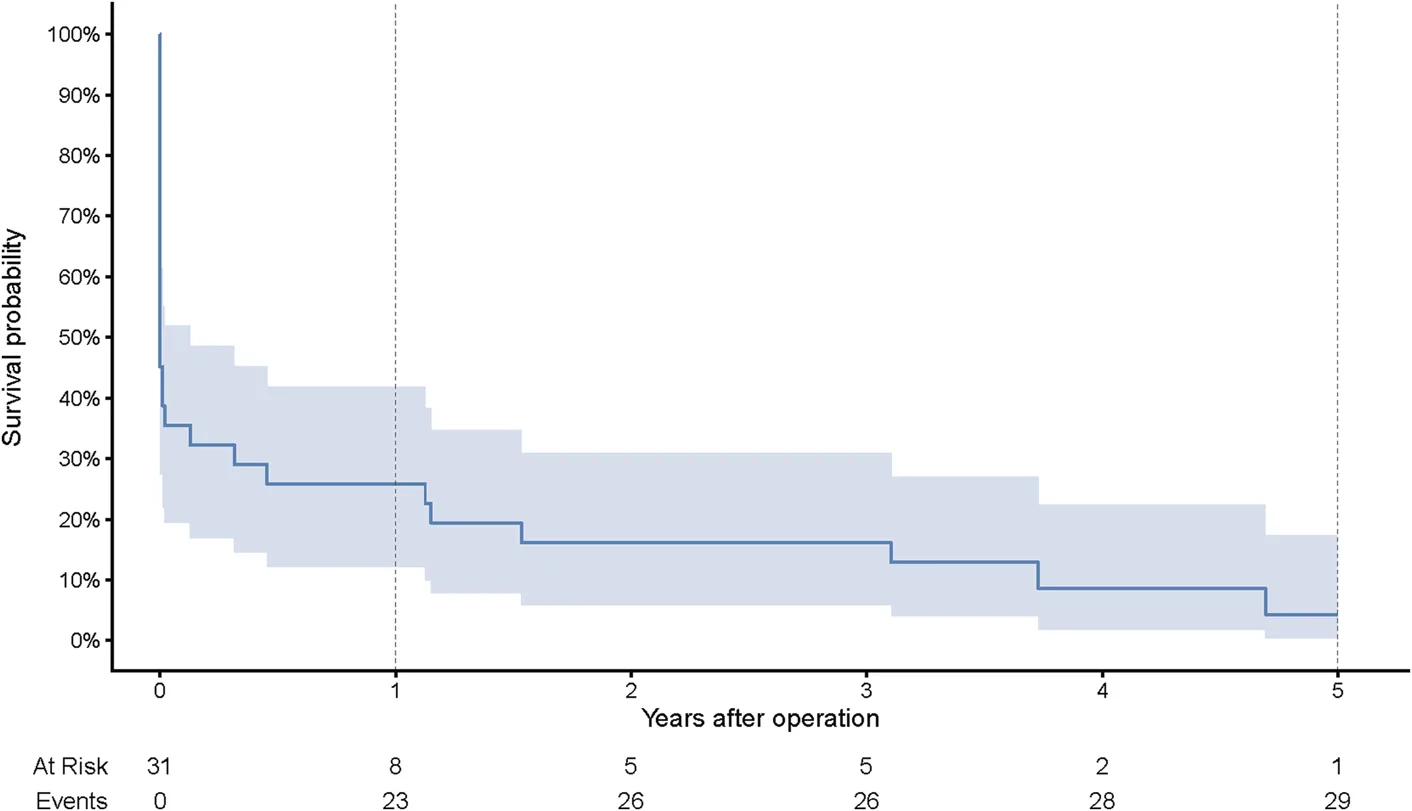
Kaplan-Meier survival curve (95% confidence interval) of all patients who experienced bone cement implantation syndrome grade 3.

Patients had a mean age of 86.2 ± 7.7 years, and 71.0% were female (Table I). Acute procedures for femoral neck fracture (FNF) accounted for 93.5% of cases, and 87.1% involved cemented prostheses. Perioperative mortality rate was 67.7%, including 17 intraoperative deaths and 4 during subsequent hospitalization. The time from surgery to death ranged from 0 to 162 hours. One- and five-year survival was 25.8% (95% CI: 12.2-41.8) and 4.3% (95% CI: 0.4-17.4), respectively (Fig. 2). No significant differences in age (87.6 vs. 83.2, p = 0.139), ASA classification (p = 0.381), sex (p = 0.105), or type of anesthesia (p = 1.000) were observed between patients who died during subsequent hospitalization and those who survived.

**Table I:** Clinical Characteristics of All Included Patients Who Experienced BCIS Grade 3

Variable	BCIS Grade 3 (n = 31)
Age at surgery in years, mean ± SD	86.2 ± 7.7
Gender, n (%)	
Female	22 (71.0)
Male	9 (29.0)
BMI in kg/m^2^, mean ± SD	23.3 ± 3.7
ASA classification, n (%)	
1	0 (0)
2	8 (25.8)
3	18 (58.1)
4	5 (16.1)
Anesthesia, n (%)	
Spinal anesthesia	11 (35.5)
General anesthesia	20 (64.5)
Procedure, n (%)	
Acute	29 (93.5)
Elective	2 (6.5)
Fixation method, n (%)	
Cemented	27 (87.1)
Uncemented	4 (12.9)
Serum hemoglobin level in mmol, mean ± SD	7.5 ± 1.1
Serum creatinine level in mmol/L, mean ± SD	114.0 ± 85.6
Medical history, n (%)	
Hypertension	16 (51.6)
Arrhythmia	17 (54.8)
COPD/asthma	4 (12.9)
Diabetes mellitus	11 (35.5)
Carcinoma	12 (38.7)
Cognitive impairment	5 (16.1)
TIA or CVA	11 (35.5)
Medication, n (%)	
Antihypertensive agents	14 (45.2)
Beta-blockers	14 (45.2)
Diuretics	16 (51.6)
Organic nitrates	4 (12.9)
Anticoagulants	8 (25.8)
Vitamin K antagonists	8 (25.8)
Antiplatelet drugs	3 (9.7)
Glucose lowering agents	8 (25.8)
Statins	13 (41.9)

ASA = American Society of Anesthesiologists, BCIS = bone cement implantation syndrome, BMI = body mass index, COPD = chronic obstructive pulmonary disease, CVA = cerebrovascular accident, and TIA = transient ischemic attack.

Among 39,237 hip procedures, 7,665 were hemiarthroplasties (19.5%), 27,847 were THA (71.0%) and 3,725 were hip revision (hemi)arthroplasties (9.5%). During hemiarthroplasty, 27 cases of BCIS grade 3 (0.4%) were observed, all of which were performed for FNF. During THA, 2 cases (0.01%) were observed, including 1 procedure performed for fracture treatment and 1 for failure of osteosynthesis material. During revision hip (hemi)arthroplasty, 2 cases (0.05%) were observed, including 1 procedure for periprosthetic fracture and 1 for failure of osteosynthesis material (Table II).

**Table II:** The Number of Identified Cases Per Hip Procedure

Procedure	Total Number n	BCIS Grade 3 n (%)
Hemiarthroplasty	7,665	27 (0.4)
THA	27,847	2 (0.01)
Revision (hemi)arthroplasty	3,725	2 (0.05)

BCIS = bone cement implantation syndrome, and THA = total hip arthroplasty.

### Hemiarthroplasties

Among 27 patients undergoing hemiarthroplasty who experienced BCIS grade 3 (Table III), 20 (74.1%) died. The mean age in this group (n = 27) was significantly higher than that in the general population undergoing hemiarthroplasty population (86.3 ± 7.7 vs. 82.4 ± 7.7 years; p = 0.009). A higher proportion of patients with BCIS grade 3 were classified as ASA IV (14.8% vs. 7.7% in the general population), although this could not be formally tested due to substantial missing data. In addition, 85.2% received a cemented hemiarthroplasty, compared with 66.8% in the general population undergoing hemiarthroplasty.

**Table III:** Clinical Characteristics of Hemiarthroplasty Procedures in Patients With BCIS Grade 3 Compared With the General Population

Variable	BCIS Grade 3 (n = 27)	General Population (n = 7,665)	p
Age at surgery, mean ± SD	86.3 ± 7.7	82.4 ± 7.7	0.009
Missing	0	339	
Sex, n (%)			0.104
Female	22 (81.5)	4,815 (65.7)	
Male	5 (18.5)	2,517 (34.3)	
Missing	0	333	
ASA score, n (%)			*
I	0	109 (1.7)	
II	7 (25.9)	1,862 (29.2)	
III	16 (59.3)	3,922 (61.4)	
IV	4 (14.8)	493 (7.7)	
Missing	0	1279	
Fixation method, n (%)			0.062†
Cemented	23 (85.2)	4,863 (66.8)	
Uncemented	4 (14.8)	2,403 (33.0)	
Hybrid	0	18 (0.2)	
Missing	0	381	

ASA = American Society of Anesthesiologists, and BCIS = bone cement implantation syndrome.

* This could not be formally tested due to substantial missing data.

† Hybrid fixation was excluded from this analysis.

Sensitivity analysis (Appendix A) showed no major differences in baseline characteristics (Appendix Table 1), number of identified cases (Appendix Table 2), clinical characteristics of hemiarthroplasty procedures compared with general population (Appendix Table 3), and one- and five-year survival rates (Appendix Figure 1).

## Discussion

This multicenter study demonstrated that BCIS grade 3 is a rare but highly lethal complication of hip arthroplasty, with an overall incidence of 0.08%. The highest incidence was observed during hemiarthroplasty for FNF (0.4%). No cases were observed during KA.

Previous studies reported BCIS grade 3 incidences ranging from 0% to 3.4%, mainly in patients undergoing hemiarthroplasty after FNF^4-16^. One larger study by Parvizi et al.^18^ reported a comparable mortality rate of 0.06% (23 of 38,488 patients). However, they excluded patients who achieved return of spontaneous circulation, thereby potentially underestimating the actual incidence of BCIS grade 3.

In this study, no cases of BCIS grade 3 were observed during KA, which is consistent with previous studies^9,19^. However, a case report by Orsini et al. (1986) described a cardiac arrest occurring during total KA following tourniquet release^20^, indicating that such events cannot be fully excluded given the retrospective design. The rarity of BCIS grade 3 in KA may be explained by several factors. KA is predominantly performed electively, whereas most BCIS cases in our study occurred during acute fracture surgery. Acute fractures disrupt bone and vascular structures, facilitating fat embolization independent of surgical manipulation^21^. Anatomical differences may also contribute, as the femoral canal is larger and more prone to cement pressurization than tibial bone, potentially generating higher embolic loads^22^. However, evidence regarding the influence of prosthesis size on BCIS risk remains conflicting^7,23^. Therefore, together with the existing literature, our data confirm that BCIS grade 3 is predominantly observed during hemiarthroplasty procedures for FNF.

The incidence of BCIS grade 3 in hemiarthroplasty (0.4%) substantially exceeds reported rates of IOCA in (non)-cardiac surgery (0.03%-0.06%)^24,25^. In addition, the mortality rate in this study (74.1%) is also higher compared with the mortality rate after perioperative cardiac arrest in general surgical populations (24.8%-58.4%). Both reflects the unique characteristics of patients undergoing hemiarthroplasty: 93.5% is acute surgery for fractures, the mean age was 87.4 years, and 16.0% were classified as ASA IV. Frailty is independently associated with increased postoperative mortality and poor outcomes after cardiopulmonary resuscitation^26-29^. Advanced age, frailty, acute physiological stress, and acute surgery are all independently associated with poor outcomes after cardiac arrest^30^. Among the survivors in our study, long-term outcomes and functional recovery data were not systematically collected, representing an important knowledge gap for future research.

Although BCIS grade 3 has traditionally been considered a complication primarily associated with cemented arthroplasty, this study identified 4 patients undergoing uncemented arthroplasty. This suggests that cementation is not the sole contributing factor to the development of severe BCIS. One possible explanation is that intramedullary pressure also increases during uncemented arthroplasty^31,32^. This rise in pressure may lead to embolic debris, although to a less prolonged and severe degree than in cemented arthroplasty^33-37^. Our finding that BCIS also occurs in cases where no cement was used further supports reconsideration of the current terminology of this syndrome. Therefore, we suggest changing it to “implant-related syndrome,” to ensure that healthcare providers remain vigilant during both cemented and uncemented procedures.

### Strengths and Limitations

This study has several limitations. First, case identification relied on a structured query, and although BCIS grade 3 would be expected to result in ICU admission or in-hospital mortality, some cases may have been missed due to non-ICU management or incomplete coding. Second, inconsistencies in clinical documentation and underreporting of cardiac arrest may have affected data completeness and accuracy. Third, relevant perioperative details, including timing of cementation, resuscitation characteristics, time to return of spontaneous circulation, neurological injury, and functional outcomes, were frequently missing from the medical records and were, therefore, not reported in this analysis. Fourth, given the retrospective design, misclassification cannot be fully excluded. Distinguishing BCIS-related cardiac arrest from other intraoperative causes, such as bleeding, anesthetic complications, or surgical events, may be challenging. Despite detailed chart review, this may have resulted in some misclassification. Fifth, several variables had more than 10% missing data. This missingness was likely not at random; therefore, formal statistical analyses were not performed to avoid biased estimates. This may have reduced the ability to detect associations. Sixth, the incidence of cemented hemiarthroplasty in this study was lower than the nationwide incidence (65.9% in 2020 according to the LROI data)^38^. This discrepancy suggests that the incidence reported in our study may not fully reflect the true national incidence of BCIS 3 in hemiarthroplasty. Seventh, the absence of data on race and ethnicity may affect the study’s generalizability.

A major strength of this study is the large sample size, allowing robust estimation of the incidence of BCIS grade 3. In addition, database queries were conducted with the support of data analysts in each participating hospital, strengthening the internal validity. Moreover, the multicenter design enhances the external validity of the findings by reflecting real-world clinical practice across different hospital settings.

## Conclusion

This large multicenter case series demonstrates that BCIS grade 3 is a rare complication (0.08%) in hip arthroplasty and was not observed in KA. The highest prevalence was found hemiarthroplasty for FNF (0.4%). These findings underscore the importance of individualized risk assessment, informed consent discussing realistic mortality risks, and consideration of alternative management strategies in very high-risk patients. We suggest changing the terminology to “implant-related syndrome,” as BCIS also occurs in cases where no cement was used. Future studies should focus on preventive strategies and long-term outcomes among survivors.

## Appendix

Supporting material provided by the authors is posted with the online version of this article as a data supplement at jbjs.org (https://links.lww.com/JBJSOA/B363). This content was not copyedited or verified by JBJS.

## References

[1] Donaldson AJ Thomson HE Harper NJ Kenny NW. Bone cement implantation syndrome. Br J Anaesth. 2009;102(1):12-22. 10.1093/bja/aen328 19059919

[2] Brokke KE Graman M Servaas S Sierevelt IN Steegers MAH Nolte PA. Bone cement implantation syndrome: a scoping review. Br J Anaesth. 2025;135(4):1038-50. 10.1016/j.bja.2025.05.041 PMC12674061 40634186

[3] Barakat N Browne JA. Is bone cement implantation syndrome actually caused by cement? A systematic review of the literature using the Bradford-Hill criteria. J Arthroplasty. 2025;40(8):S353-9. 10.1016/j.arth.2025.01.039 39884480

[4] Kaufmann KB Baar W Rexer J Loeffler T Heinrich S Konstantinidis L Buerkle H Goebel U. Evaluation of hemodynamic goal-directed therapy to reduce the incidence of bone cement implantation syndrome in patients undergoing cemented hip arthroplasty - a randomized parallel-arm trial. BMC Anesthesiol. 2018;18(1):63. 10.1186/s12871-018-0526-4 PMC5991443 29875024

[5] Jaffe JD Edwards CJ Hamzi R Khanna AK Olsen F. Bone cement implantation syndrome: incidence and associated factors in a United States setting. Cureus. 2022;14(11):e31908. 10.7759/cureus.31908 PMC9792331 36579204

[6] Weingärtner K Störmann P Schramm D Wutzler S Zacharowski K Marzi I Lustenberger T. Bone cement implantation syndrome in cemented hip hemiarthroplasty—a persistent risk. Eur J Trauma Emerg Surg. 2022;48(2):721-9. 10.1007/s00068-020-01587-8 PMC9001528 33495852

[7] Chulsomlee K Prukviwat S Tuntiyatorn P Vasaruchapong S Kulachote N Sirisreetreerux N Tanphiriyakun T Chanplakorn P Sa-ngasoongsong P. Correlation between shape-closed femoral stem design and bone cement implantation syndrome in osteoporotic elderly femoral neck fracture undergoing cemented hip arthroplasty: a retrospective case-control study in 128 patients. Orthop Traumatol Surg Res. 2023;109(1):103450. 10.1016/j.otsr.2022.103450 36273503

[8] Olsen F Kotyra M Houltz E Ricksten SE. Bone cement implantation syndrome in cemented hemiarthroplasty for femoral neck fracture: incidence, risk factors, and effect on outcome. Br J Anaesth. 2014;113(5):800-6. 10.1093/bja/aeu226 25031262

[9] Rassir R Schuiling M Sierevelt IN van der Hoeven CWP Nolte PA. What are the frequency, related mortality, and factors associated with bone cement implantation syndrome in arthroplasty surgery?. Clin Orthop Relat Res. 2021;479(4):755-63. 10.1097/CORR.0000000000001541 PMC8083844 33165048

[10] Bhadani JS Kumar I Ahmed W Akhileshwar A Kumar S Kumar A. Hemodynamic effects of bone cement implantation in hip arthroplasty: insights from a prospective study in eastern India. J Orthop Case Rep. 2024;14(8):212-21. 10.13107/jocr.2024.v14.i08.4702 PMC11327671 39157488

[11] Fernandez MA Henshaw F Carlos WJ Kelly A Griffin XL Costa ML. Haemodynamic measurements during hup hemiarthroplasty surgery for hip fracture. Bone Joint J. 2025;107-B(1):103-7. 10.1302/0301-620X.107B1.BJJ-2024-0548.R1 39740680

[12] Zastrow RK Rao SS Morris CD Levin AS. The effect of anesthetic regimen on bone cement implantation syndrome in cemented hemiarthroplasty for hip fracture. J Am Acad Orthop Surg. 2025;33(1):e46-e57. 10.5435/JAAOS-D-24-00239 39231275

[13] Ukaj S Veslko M Krasniqi S Podvorica V Ukaj F Ahmeti A Hernigou P Cimerman M. Cemented stems in healthy elderly patients result in higher hypoxia despite a paradoxical lower femoral increase of intramedullary pressure. Int Orthop. 2021;45(4):915-22. 10.1007/s00264-021-04955-0 33528632

[14] Yuenyongviwat V Janejaturanon J Hongnaparak T Iamthanaporn K. Comparative assessment of bone cement implantation syndrome in cemented bipolar hemiarthroplasty: impact in patients with and without preexisting heart disease. Orthop Rev. 2024;16:122320. 10.52965/001c.122320 PMC11364534 39219732

[15] García-Mansilla A Castro Lalín A Holc F Molho NM Vescovo A Slullitel PA Buttaro MA. Intraoperative unfractionated heparin before femoral component cementation should be avoided in femoral neck fracture treated with hybrid total hip arthroplasty. Eur J Orthop Surg Traumatol. 2023;33(6):2547-54. 10.1007/s00590-023-03472-7 36645495

[16] Miyamoto S Nakamura J Iida S Shigemura T Kishida S Abe I Takeshita M Harada Y Orita S Ohtori S. Intraoperative blood pressure changes during cemented versus uncemented bipolar hemiarthroplasty for displaced femoral neck fracture: a multi-center cohort study: the effect of bone cement for bipolar hemiarthroplasty in elderly patients. Arch Orthop Trauma Surg. 2017;137(4):523-9. 10.1007/s00402-017-2651-9 28213848

[17] Landelijke Registratie Orthopedische Interventies. Completeness. 2026. Available at: https://www.lroi.nl/jaarrapportage/data-quality/completeness/. Accessed February 13 2026.

[18] Parvizi J Holiday AD Ereth MH Lewallen DG. The Frank Stinchfield Award. Sudden death during primary hip arthroplasty. Clin Orthop Relat Res. 1999;369:39-48. 10.1097/00003086-199912000-00005 10611859

[19] Dumanlı Özcan AT Kesimci E Balcı CA Kanbak O Kaşıkara H But A. Comparison between colloid preload and coload in bone cement implantation syndrome under spinal anesthesia: a randomized controlled trial. Anesth Essays Res. 2018;12(4):879-84. 10.4103/aer.AER_127_18 PMC6319061 30662124

[20] Orsini EC Richards RR Mullen JM. Fatal fat embolism during cemented total knee arthroplasty: a case report. Can J Surg. 1986;29(5):385-6. 3756668

[21] Timon C Keady C Murphy CG. Fat embolism syndrome – a qualitative review of its incidence, presentation, pathogenesis and management. Malays Orthop J. 2021;15(1):1-11. 10.5704/MOJ.2103.001 PMC8043637 33880141

[22] Gozzard C Gheduzzi S Miles AW Learmonth ID. An in-vitro investigation into the cement pressurization achieved during insertion of four different femoral stems. Proc Inst Mech Eng H. 2005;219(6):407-13. 10.1243/095441105X34400 16312100

[23] Schwarzkopf E Sachdev R Flynn J Boddapati V Padilla RE Prince DE. Occurrence, risk factors, and outcomes of bone cement implantation syndrome after hemi and total hip arthroplasty in cancer patients. J Surg Oncol. 2019;120(6):1008-15. 10.1002/jso.25675 PMC7357733 31432531

[24] Kaiser HA Saied NN Kokoefer AS Saffour L Zoller JK Helwani MA. Incidence and prediction of intraoperative and postoperative cardiac arrest requiring cardiopulmonary resuscitation and 30-day mortality in non-cardiac surgical patients. PLoS One. 2020;15(1):e0225939. 10.1371/journal.pone.0225939 PMC6975552 31967987

[25] Fielding-Singh V Willingham MD Fischer MA Grogan T Benharash P Neelankavil JP. A population-based analysis of intraoperative cardiac arrest in the United States. Anesth Analgesia. 2020;130(3):627-34. 10.1213/ANE.0000000000004477 31651456

[26] Allen MB Orkaby AR Justice S Hall DE Hu FY Cooper Z Bernacki RE Bader AM. Frailty and outcomes following cardiopulmonary resuscitation for perioperative cardiac arrest. JAMA Netw Open. 2023;6(7):e2321465. 10.1001/jamanetworkopen.2023.21465 PMC10318473 37399014

[27] Dayama A Olorunfemi O Greenbaum S Stone ME McNelis J. Impact of frailty on outcomes in geriatric femoral neck fracture management: an analysis of national surgical quality improvement program dataset. Int J Surg. 2016;28:185-90. 10.1016/j.ijsu.2016.02.087 26926088

[28] Moppett IK Kane AD Armstrong RA Kursumovic E Soar J Cook TM; NAP7 Panel and Team Members. Peri-operative cardiac arrest in the older frail patient as reported to the 7th National Audit Project of the Royal College of Anaesthetists. Anaesthesia. 2024;79(8):810-20. 10.1111/anae.16267 38556808

[29] Hamlyn J Lowry C Jackson TA Welch C. Outcomes in adults living with frailty receiving cardiopulmonary resuscitation: a systematic review and meta-analysis. Resusc Plus. 2022;11:100266. 10.1016/j.resplu.2022.100266 PMC9256816 35812717

[30] Göbel Andertun S Wissendorff-Ekdahl A Ullén S Cronberg T Friberg H Jakobsen JC Blennow Nordström E Heimburg K Cariou A Grejs AM Haenggi M Hovdenes J Robba C Rylander C Taccone FS Wise MP Young PJ Nielsen N Lilja G. Impact of frailty on mortality, functional outcome, and health status after out-of-hospital cardiac arrest: insights from the TTM2-trial. Intensive Care Med. 2025;51(12):2367-77. 10.1007/s00134-025-08185-5 PMC12678506 41212202

[31] Hofmann S Hopf R Mayr G Schlag G Salzer M. In vivo femoral intramedullary pressure during uncemented hip arthroplasty. Clin Orthopaedics Relat Res. 1999;360:136-46. 10.1097/00003086-199903000-00017 10101319

[32] Smith PN Leditschke A McMahon D Sample RR Perriman D Prins A Brüssel T Li RW. Monitoring and controlling intramedullary pressure increase in long bone instrumentation: a study on sheep. J Orthop Res. 2008;26(10):1327-33. 10.1002/jor.20564 18464262

[33] Christie J Robinson CM Singer B Ray DC. Medullary lavage reduces embolic phenomena and cardiopulmonary changes during cemented hemiarthroplasty. J Bone Joint Surg Br Vol. 1995;77-B(3):456-9. 7744936

[34] Christie J Burnett R Potts HR Pell AC. Echocardiography of transatrial embolism during cemented and uncemented hemiarthroplasty of the hip. J Bone Joint Surg Br Vol. 1994;76-B(3):409-12. 8175843

[35] Pitto RP Koessler M Kuehle JW. Comparison of fixation of the femoral component without cement and fixation with use of a bone-vacuum cementing technique for the prevention of fat embolism during total hip arthroplasty. A prospective, randomized clinical trial. J Bone Joint Surg. 1999;81(6):831-43. 10.2106/00004623-199906000-00010 10391548

[36] Bisignani G Bisignani M Pasquale GS Greco F. Intraoperative embolism and hip arthroplasty: intra-operative transesophageal echocardiographic study. J Cardiovasc Med. 2008;9(3):277-81. 10.2459/JCM.0b013e32807fb03a 18301146

[37] Hagio K Sugano N Takashina M Nishii T Yoshikawa H Ochi T. Embolic events during total hip arthroplasty: an echocardiographic study. J Arthroplasty. 2003;18(2):186-92. 10.1054/arth.2003.50027 12629609

[38] Duijnisveld BJ Koenraadt KLM van Steenbergen LN Bolder SBT. Mortality and revision rate of cemented and uncemented hemiarthroplasty after hip fracture: an analysis of the Dutch Arthroplasty Register (LROI). Acta Orthop. 2020;91(4):408-13. 10.1080/17453674.2020.1752522 PMC8023919 32285730

